# Lockdown in a specialised rehabilitation unit: the best of times

**DOI:** 10.1017/ipm.2020.50

**Published:** 2020-05-21

**Authors:** D. Glancy, L. Reilly, C. Cobbe, M. Glynn, S. Punchoo, K. Foy

**Affiliations:** National Specialised Rehabilitation Unit, Bloomfield Hospital, Dublin, Ireland

**Keywords:** Psychotherapy group, rehabilitation, schizophrenia, therapeutic community, trauma

## Abstract

Specialised rehabilitation units offer inpatient multi-disciplinary rehabilitation for individuals with severe and enduring mental illness. A cornerstone of therapy is the work in the community through further education and community organisations. However, coronavirus restrictions have meant that such external supports are no longer available for the duration of the crisis. This has led to opportunities for developing new ways of offering rehabilitation within hospital environments. This article describes some of the new initiatives developed. The benefits of the lockdown for service users are also discussed. Many found the cessation of visits from family members with whom they had an ambivalent relationship helpful. The lockdown improved relationships between patients on the unit and encouraged a greater feeling of community. The lockdown has also emphasised the importance of team self-awareness and an awareness of the nature of the treatments offered.

Bravery, courage, love and care,

*Stop many a tear* (Day, 2019)

## Introduction

The National Mental Health Division established placements at Specialised Rehabilitation Units for individuals with severe chronic and enduring mental health illnesses at Bloomfield Hospital, Dublin (HSE Mental Health Services, 2018). The patients referred to the unit typically have a history of complex treatment refractory psychiatric symptoms and multiple prolonged admissions to acute mental health units. They have reduced ability to manage in the community despite intensive management from their local community mental health teams. The service was only established in 2018 and since then has accepted referrals from all parts of the country. Whilst the majority of the service users have a diagnosis of schizophrenia, all have additional mental health needs and most have a history of complex trauma. Our multidisciplinary team consists of specialists from psychiatry, psychology, occupational therapy, nursing as well as a peer support worker. The rehabilitation offered is individualised, goal orientated and led by service user-generated goals.

Prior to the national coronavirus-19 emergency, a fundamental pillar of the rehabilitation offered was an emphasis on activities in the community and outside the unit. As a result, our clients attended a variety of local services including adult education, men’s sheds group, tidy towns groups, voluntary work in charity shops and fitness activities in local sport and leisure facilities.

The lockdown restrictions meant that such local supports and activities were no longer available. The Multidisiplinary Team (MDT) in conjunction with service users therefore had to develop additional activities to support the rehabilitative programme.

## Family: a double bind

Previously, most patients on the unit had weekly visits from family members. These visits were generally perceived to be helpful, and many of the patients had close relationships with their relatives.

As family contact was curtailed due to the lockdown, many service users felt better able to reflect and empowered to speak with therapists about the nature of those relationships. Familial constellation and the role of the patient within that system became much more apparent. Patients opened up more during psychotherapeutic sessions about significant trauma or attachment issues. Trauma and attachment issues within families can contribute to high levels of unhealthy enmeshment creating chaotic boundaries, difficulties with emotional regulation and poor sense of self for the individuals. One of the service users summarised the new world of the lockdown as ‘*no visits, no calls – peace and space*’.

Whilst families may be supportive, the family can also be the source of trauma triggers for those with complex trauma history. As stated by Aldersey & Whitley (2015), ‘*families both facilitate and impede recovery process*’.

The lockdown created an opportunity to explore these challenges in a safe and non-threatening environment during 1:1 sessions and group sessions. The reduced visits from relatives meant that patients felt better able to examine patterns of communication, relationships, power structures and other aspects of family systems.

## Reduced self-expectations

Many of the service users are highly self-critical, perfectionistic and have experienced considerable rejection and perceived failure throughout their time in psychiatric services. One of our patient summarised this as ‘*my doctor said that there was nothing he could do for me…I’m not like the others*’. In essence, they have been through a revolving door with multiple relapses and readmissions. During the COVID-19 pandemic, they described their setting as ‘*safe*’ and that ‘*everyone is in the same boat*’. They reported feeling less expectations being placed on them, both by themselves and others. The team noted that it was easier to collaboratively work on development of grounding techniques, addressing internal critic, practising mindfulness, exploring past trauma and integrating self, improving self-care and improving awareness of emotions.

## Service user reflection

Prior to the restrictions, self-reflection could be easily avoided through engagement in a myriad of distraction and avoidance techniques. Getting in touch with internal processes to integrate mind and body awareness has been an enriching though difficult journey for patients. For most, self-awareness improved, and there was increased hope for the future by addressing the past with one of our clients saying ‘*I am fearful but hopeful that I will see the light at the end of the tunnel*’.

During the lockdown, some reflected on their rehabilitation journey and one stated ‘*I can’t believe I was allowed out on my own, and now I appreciate it even more, I will make full use of it in the future*’.

According to Herman (1997), ‘*safety, remembering, mourning and reconnection are essential trauma resolution preambles*’ which summarise the self-reflection for many during the lockdown.

## Service self-reflection

With the onset of COVID-19, previous routines have been thrown into chaos. The service has now adapted the therapeutic programme to enable this period to be a learning experience of self-discovery and examine our values, beliefs and raison d’etre. The focus has shifted to increasing group-based activities on the ward and looking at innovative ways to occupy this time based on service user needs.

The team has committed to provision of trauma-focused therapy and developed educational processes with staff psychology book clubs and lunchtime education groups.

## Service adaptations

A tai chi group is delivered daily on the unit focusing on grounding, breathing and self-soothing (Kong *et al*. 2019). The group is attended by everyone involved in the unit, staff and service users alike. A ‘time to talk’ group was established within the service to address social and relationship skills for service users with co-morbid learning disability or developmental disorders. The group was designed as an open forum to explore gender identity, expression and sexuality in the context of their own lives and society as a whole. A separate psychotherapy group was established for individuals with higher level functioning using the Yalom model (Yalom & Lesczc, 2005).

The ethos of the Wellness Recovery Action Plan (WRAP) (Copeland, 2011) and the decider skills group (Ayres & Vivyan, 2019) fosters personal responsibility, developing coping skills and use of grounding techniques. The delivery of other existing group programmes has also undergone changes with the music therapist now delivering their sessions over the Zoom application. An on-site greenhouse has afforded the opportunity for one service user to lead a gardening segment to fellow interested service users called ‘how’s it growing’.

## Developing a therapeutic community

There are increasing tangible examples of shared camaraderie evident on the unit including service users sharing their cooked food and blossoming friendships among others. While some of these interactions have occurred spontaneously, the unit itself has focused on creating a therapeutic community with organisation of activities such as afternoon tea and outdoor hikes within the 2 km distance. Like the rest of the country, time was spent to enhance the physical environment by improving the garden and outdoor spaces with planting. The focus of this was to generate a common purpose and ownership of the shared space through engagement in meaningful pursuits. However, within any confined space, it is anticipated that conflict will occur, and healthy outbursts have been welcomed as individuals are encouraged to share how they openly feel about situations and resolve it accordingly.

At the weekly peer support group, residents were offered the opportunity to express their thoughts and feelings about the restrictions in place as a result of the coronavirus outbreak.

Despite the increased focus on group and 1:1 sessions, maintaining social contact with the outside world has remained pivotal. Service users were offered the same rights as everyone else to access the community in line with the national lockdown regulations. Service users can access essential services within the community such as post office, bank and essential shops. A preventative approach to COVID-19 was adopted with emphasis placed on hand hygiene and social distancing measures and adapted education sessions for this were developed and delivered.

Wilcock (2002) discusses the theory of ‘*doing, being and becoming*’ as central components for achieving wellness and realising self-actualisation. As a service in its infancy, this period of lockdown has encapsulated a greater balance between the three aspects, moving from primarily doing to being and becoming. As the psychiatrist Victor Frankl (1984) once said, ‘*when we are no longer able to change a situation, we are challenged to change ourselves*’.

## Ending the lockdown

The team believes that the end of the lockdown shall present its own challenges. The possibility of reinstatement of contact with family members, returns to activities in the community and increased external distractions have the potential to be testing. As a service, we plan to devote a number of group therapy and individual sessions to reflect on the past number of months and the return to a state of normality. Using questionnaires, we hope to assess the service users and staff attitudes to the changes made to the programme during the lockdown. The results of these questionnaires along with wider patient and staff discussions will inform how we integrate the lockdown programme into a post-lockdown world.

## Conclusion

The lockdown allowed the team the space and opportunity to self-reflect on the essence of what defines our work. It allowed both service users and staff the opportunity to reflect on the shared journey that we are taking together and the necessity for collaboration, honesty and open dialogue. Whilst families can often be seen as an important resource for service users, we became more aware of the double-edged nature of family relationships – particularly in individuals who have traumatic, ambivalent or challenging relationships with their relatives. All too often, in healthcare, we tend to prioritise action at the expense of reflection. The COVID-19 emergency allowed this service to challenge that and to instead focus on developing a true therapeutic community from within.

## Conflict of interest

None.

## Ethical standards

The authors assert that all procedures contributing to this work comply with the ethical standards of the relevant national and institutional committee on human experimentation with the Helsinki Declaration of 1975, as revised in 2008. The authors assert that ethical approval for publication of this manuscript.
